# Management of Advanced Squamous Cell Carcinoma of the Vulva

**DOI:** 10.3390/cancers14010167

**Published:** 2021-12-30

**Authors:** Linda J. Rogers

**Affiliations:** 1 Department of Obstetrics and Gynaecology, Groote Schuur Hospital, The University of Cape Town, Cape Town 7505, South Africa; linda.rogers@uct.ac.za; 2 SAMRC/UCT Gynaecological Cancer Research Centre, University of Cape Town, Cape Town 7925, South Africa

**Keywords:** advanced vulvar cancer, squamous carcinoma, chemoradiation, targeted therapies

## Abstract

**Simple Summary:**

Vulvar cancer is a rare gynaecological malignancy that has an increasing incidence, particularly in younger women. Early vulvar cancer can be treated and cured with surgical excision. Approximately 30% of women present with advanced disease, which requires treatment either with mutilating surgery or a combination of chemotherapy and radiotherapy, which is an effective treatment but has many side effects. Current research is focused on new less morbid approaches to treatment, in which drugs that target various steps on the biological pathway from pre-cancer to cancer are used, with the aim of preventing the growth of vulvar cancers. This review is an update of the current management of women with advanced vulvar cancer.

**Abstract:**

Vulvar cancer is a rare gynaecological malignancy, accounting for 2–5% of cancers of the female genital tract. Squamous cell carcinoma is the most frequently occurring subtype and, historically, has been a disease of older post-menopausal women, occurring with a background of lichen sclerosus and other epithelial conditions of the vulvar skin that may be associated with well-differentiated vulvar intra-epithelial neoplasia (dVIN). An increase in human papillomavirus (HPV) infections worldwide has led to an increase in vulvar squamous carcinomas in younger women, resulting from HPV-associated high-grade vulvar squamous intra-epithelial lesions (vHSIL). Surgical resection is the gold standard for the treatment of vulvar cancer. However, as approximately 30% of patients present with locally advanced disease, which is either irresectable or will require radical surgical resection, possibly with a stoma, there has been a need to investigate alternative forms of treatment such as chemoradiation and targeted therapies, which may minimise the psychosexual morbidity of radical surgery. This review aims to provide an update on management strategies for women with advanced vulvar cancer. It is hoped that investigation of the molecular biologies of the two different pathways to vulvar squamous cell carcinoma (HPV-associated and non-HPV-associated) will lead to the development of targeted therapeutic agents.

## 1. Introduction

Vulvar cancer is a rare gynaecological malignancy and accounts for approximately 5% of all cancers of the female genital tract [1]. Squamous cell carcinoma is the most frequently occurring subtype and, historically, has been regarded as a disease of older and post-menopausal women, occurring with a background of lichen sclerosus and other epithelial conditions of the vulvar skin that may be associated with well-differentiated vulvar intra-epithelial neoplasia (dVIN) [2].

More recently, however, an increase in human papillomavirus (HPV) infections worldwide has led to an increase in squamous carcinomas of the vulva in younger women, resulting from HPV-associated high-grade vulvar squamous intra-epithelial lesions (vHSIL). HPV is thought to be the cause of 40% of all vulvar squamous carcinomas [3].

The International Federation for Gynaecology and Obstetrics (FIGO) staging system for vulvar cancer has recently been updated (see Table 1) [4]. The term “Advanced Vulvar Cancer” may be considered to include very large or locally advanced tumours (stage I and II with tumours >4 cm in diameter); those with macroscopic nodal metastases (stage IIIB and IIIC); those that involve the urethra, bladder, anorectum, or pelvic bone (stage IVA); those for which adequate surgical excision requires sacrifice of the urethra or anus or an exenterative procedure; and those with distant metastases (stage IVB). About 30–40% of women with vulvar cancer present with advanced stage tumours [5]. These often result from late presentation to the appropriate health care services, and the reasons for this may include patient factors such as older age and embarrassment; educational deficiencies on the part of both healthcare professionals and patients; and lack of access to medical care, particularly in less well-resourced areas of the world.

The definition of the term “locally advanced vulvar cancer” (LAVC) is controversial. In their review of anovulvectomy and its alternatives, O’Donnell et al. define LAVC as cancers that encroach upon or cross the borders with surrounding structures such as the urethra or anus” [6]. Aragona and his colleagues authored a paper on defining the concept of LAVC, and they defined it as “a clinical presentation of the disease, without distant metastasis, when primary treatment with radical surgery is not feasible due to the presence of unresectable disease; that is, the impossibility to remove the tumor with adequate surgical margins, and consequently the need to use neoadjuvant therapy, primary CCHR [concurrent chemoradiotherapy], or else, ultraradical surgery. Inguinofemoral nodes are included in this definition when they are fixed to fascia, muscle, or vascular structures” [7].

Surgical resection is the gold standard for the treatment of vulvar cancer [6]. However, as approximately one-third of vulvar cancer patients present with locally advanced disease, which is either irresectable or will require radical surgical resection, possibly with a stoma, there has been a need to investigate alternative forms of treatment such as chemoradiation and targeted therapies, which may minimise the psychosexual morbidity of radical surgery.

The aim of this review is to provide an update on management strategies for women with advanced vulvar cancer, with particular emphasis on research into new modalities of treatment. There have been few new proven therapies for women with advanced or recurrent disease in the past few decades, and it is hoped that investigation of the molecular biologies of the two different pathways to vulvar squamous cell carcinoma (HPV-associated and non-HPV-associated) will lead to the development of targeted therapeutic agents.

## 2. Diagnosis, Staging, and Investigations

Histological confirmation is needed before treatment is planned. Many women with locally advanced disease have a lot of pain and discomfort, and incisional biopsy in theatre, along with examination under anaesthesia, is often helpful in order to confirm the diagnosis and determine the local extent of disease.

Women with clinically suspicious groin nodes should have a fine needle aspiration (FNA) or biopsy of the nodes in cases where node positivity would change primary treatment [8,9]. Abdomen and pelvis CT, MRI or PET-CT may help to determine the extent of nodal involvement in the groin, pelvic, and para-aortic nodes, and whether there are any distant metastases [10]. It is particularly important to exclude distant metastases when ultraradical surgery is planned.

## 3. Management

The management of women with advanced vulvar cancer is complex and should be undertaken by a multidisciplinary team in a Gynaecological Oncology Centre. Gynaecological oncologists, clinical oncologists, psychologists, dieticians, radiotherapists, clinical nurse specialists, palliative care practitioners and others are vital members of the team. Plastic surgeons can assist with vulvar reconstruction procedures, and colorectal surgeons can assist with laparoscopic stoma formation where appropriate and depending on local expertise. Resources permitting, patients should be referred to a lymphoedema service prior to treatment.

Ideally, the status of the patient’s groin lymph nodes should be determined first, as this will assist in planning their treatment [10]. If there are no suspicious nodes clinically and on imaging, then bilateral inguinofemoral lymphadenectomies can be performed with or without radical wide local excision of the vulvar primary (or radical vulvectomy) if this is resectable.

## 4. Treatment Options

### 4.1. Surgery

#### 4.1.1. Primary Tumour

Radical wide local excision of the vulval tumour, where possible, is the gold standard of treatment, though not for stage IV tumours (with involvement of bone, fixed nodes, or distant metastases). In the latter, the role of surgery is limited to palliation of symptoms.

The type of surgery is individualised to the particular patient, with reference to the size and location of the tumour [8]. For most locally advanced/large cancers, this will involve a radical wide local excision or radical vulvectomy and inguinofemoral lymphadenectomies via separate incisions. Where there are enlarged or fixed lymph nodes, the risk of recurrence in the skin bridge is higher, and an en-bloc radical vulvectomy and groin dissection may be considered [11]. Radical vulvectomy may be preferred over radical wide local excision if there is extensive pre-invasive disease present, so that this “field of cancerisation”, a potential site of re-occurrence of tumour, can also be excised [12].

The aim of surgery is to remove the tumour with a surgical margin of 1–2 cm and a pathological margin of at least 8 mm [8], though a recent systematic review has found that margins of <8 mm are not associated with increased risk of recurrence [13]. In larger tumours, part of the lower vagina or distal urethra may need to be included in the resection in order to achieve adequate/tumour-free margins. If primary closure is not possible, closure may be by means of a flap, or the wound can be left to heal by secondary intention. There are a wide variety of both local and distant flaps that enable wound healing after radical excision of vulvar tumours and reduce the side effects of vulvar scarring [8].

A colostomy may be required as a temporary measure in order to facilitate healing of a flap, or as part of an anovulvectomy or posterior exenteration, if the anus and rectum are removed as part of the surgery.

Other techniques such as neoadjuvant chemotherapy or primary chemoradiation may be employed, the former to attempt to shrink the tumour enough to allow surgical resection without compromising the anus or urethra and the latter as an alternative definitive treatment to exenteration.

In advanced/stage IV disease, the role of surgery is palliative: urinary diversions and/or defunctioning stomas can be used to improve quality of life by controlling the unpleasant symptoms of fistulae.

#### 4.1.2. Groin Lymph Nodes

Inguinofemoral lymphadenectomy is the most important way of treating and assessing the groins of women with multifocal vulvar tumours, or those that are greater than 4 cm in size. When there are enlarged (greater that 2 cm in size) metastatic nodes, debulking is important, as radiotherapy is not usually able to sterilise metastases greater than 2 cm [9]. “Debulking” rather than a full groin dissection is recommended, as these patients will require post-operative radiotherapy and are likely to have less morbidity from debulking and radiotherapy than after a full groin dissection followed by radiotherapy [14,15].

#### 4.1.3. Outcomes and Complications of Surgery

There is a high rate of complications following inguinofemoral lymphadenectomy. The incidence of wound breakdown is about 34%, and about 25–45% of women develop lymphocysts and/or lymphoedema [16,17]. There is a lack of evidence to support any of the strategies to decrease the complication rate, though preservation of the long/great saphenous vein is recommended, as this is thought to reduce the incidence of cellulitis and lymphoedema [18]. Suction drains are usually inserted into the groin wounds, though the optimal management of groin wound drainage is not yet known [19].

The incidence of lymphoedema is substantially greater in women who are treated with both surgery and radiotherapy [20], and prevention of lymphoedema should be discussed with patients prior to treatment. Ideally, these women should be referred to a specialist lymphoedema service for further care [8].

#### 4.1.4. Indications for Post-Operative (Chemo)Radiotherapy

Adjuvant or post-operative (chemo)radiation should be considered when:

- There are positive excision margins of the vulval tumour, and re-excision is not possible;
- Pathological margins are less than 2 mm (associated with increased rates of local recurrence) [9], and no re-excision is possible; and
- There is more than one lymph node that contains metastases and/or there is extracapsular spread of tumour [8].

External beam radiotherapy is usually given, ideally within 6 weeks of surgery if being used in the adjuvant setting. It is delivered by intensity modulated radiation therapy (IMRT) and volumetric modulated arc therapy (VMAT), and CT or MRI planning are used to enable the target volume to be calculated very precisely and to minimise the dose of radiation to surrounding organs such as the bladder, rectum and small bowel, and the femoral heads [8].

The use of chemotherapy (most commonly Cisplatin) as a radiation sensitiser, given concomitantly with adjuvant radiation for vulvar cancer, is supported mainly by evidence extrapolated from the treatment of squamous carcinomas of the cervix, anus, and head and neck [9]. Analysis of population data has shown that the addition of chemotherapy to adjuvant radiation reduced the risk of death by 38% (HR 0.62, 95% CI 0.48–0.79, *p* = 0.001) [21].

### 4.2. Chemoradiation with or without Subsequent Surgery

Several reports of the use of pre-operative radiotherapy to “debulk” tumours that would otherwise have required exenterative treament suggested that radiotherapy could be used to reduce the extent of surgery or even eliminate the need for surgery completely if a high enough dose of radiotherapy was used [22,23,24]. When it was observed that the use of chemotherapy with mitomycin-C and 5-fluorouracil concomitantly with radiation treatment resulted in long-term control and even cure of squamous carcinomas of the anus, chemoradiation was investigated as a primary therapy for vulvar cancer. Thomas et al. reported complete response rates of 66% in patients receiving chemoradiation [24].

A 2006 Cochrane review by Van Doorn et al. [25], updated in 2011 [26], sought to evaluate the effectiveness and safety of neoadjuvant and primary chemoradiation for women with locally advanced primary vulval cancer compared to other primary modalities of treatment such as primary surgery or primary radiation. The initial review included five studies, though none of them were randomised controlled trials (RCT): Eifel 1995 [27], Landoni 1996 [28], Montana 2000 [29], Moore 1998 [30], and Scheistron 1993 [31]. The rationale of neoadjuvant chemoradiation was reduction in the primary tumour to optimise surgical resection, while also treating micrometastases. The most frequently described radiosensitisers were cisplatin, 5-fluorouracil, and mitomycin C. Operability was achieved in 63–92% of cases, and survival was improved if the residual tumour was resected. There were, however, severe mucocutaneous side-effects, which are improved with modern IMRT techniques [25]. The review concluded that the complications of chemoradiation might outweigh the complications of exenteration, and that “neoadjuvant therapy is not justified in patients with tumours that can be adequately treated with radical vulvectomy and bilateral groin node dissection alone” [25].

In the updated review [26], one RCT and two non-randomised studies that allowed for multivariate analyses met the inclusion criteria and included a total of 141 women. This review concluded that women with advanced vulvar cancer had no difference in overall survival or treatment-related adverse events when chemoradiation was compared with primary surgery. There was a high risk of bias due to a wide variety of chemoradiation regimens, lack of standard terminology for “operable and inoperable vulval cancer”, and the lack of quality of life data [24].

Definitive chemoradiation is, however, the treatment of choice in disease that is irresectable without radical surgery, either because the patient does not wish to have exenterative surgery and a stoma, or because the patient is unfit for major surgery and anaesthesia.

Doses of at least 60 Gy are administered: initially, 45–50 Gy to the pelvis and groins, and then an additional 15–20 Gy photon or electron boost to “gross disease”. An interstitial implant or IMRT can also be used to deliver a selective boost to residual disease on the vulva [8]. Concomitant cisplatin chemotherapy at a dose of 40mg/m^2^ should also be given weekly [32].

### 4.3. Palliative Radiotherapy

This may be used at initial presentation of locally advanced disease, when other treatment options are not appropriate, or to treat a recurrence that is irresectable. It can be used to relieve symptoms such as bleeding and pain and is given in short courses of one to two weeks, using 20 Gy in 5 fractions or 30 Gy in 10 fractions [8].

### 4.4. Neoadjuvant Chemotherapy

Chemotherapy has an important and evolving role in the management of advanced vulvar cancer. It may be used:

1. As a neoadjuvant treatment to downstage disease prior to surgery, thus avoiding exenteration;
2. With concomitant radiation after surgery or as a primary treatment; and
3. To treat recurrent and metastatic disease.
4. Neoadjuvant chemotherapy (NACT) may also be used to treat women with vulvar cancer who are too unfit for radical surgery or radiation.

As the role of NACT in the treatment of advanced vulvar cancer is not yet well defined, a large prospective multicentre study is required to evaluate the outcomes of women treated with neoadjuvant chemotherapy-response rates have as yet only been reported in small case series. The advantages of NACT, such as establishing whether the tumour is chemo-sensitive or not, potential treatment of subclinical metastases, and lower risk of perioperative complications than after radiotherapy, are not inconsiderable.

**Figure 1:**
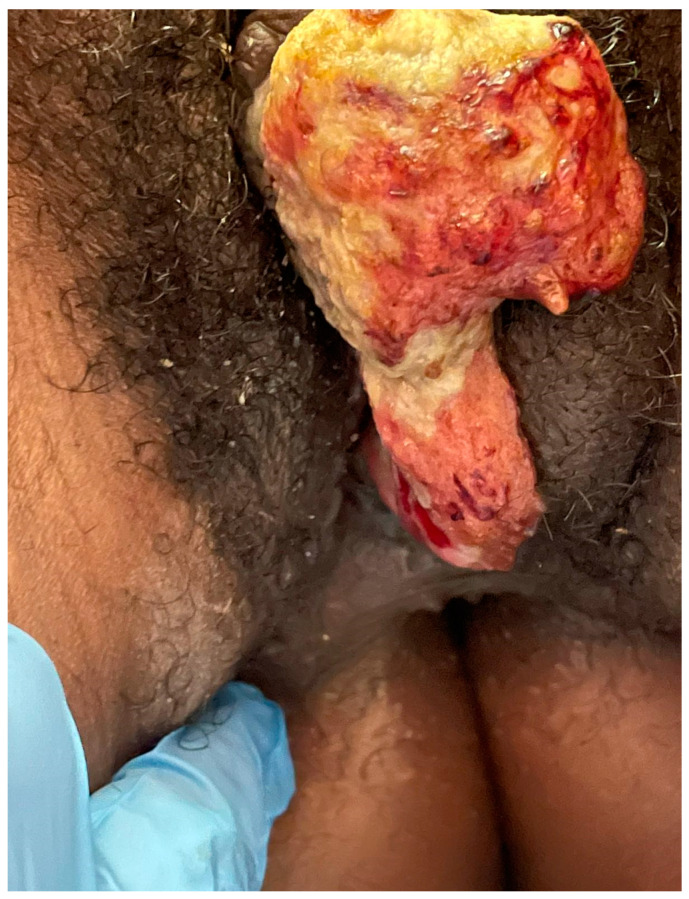
Prior to treatment.

**Figure 2:**
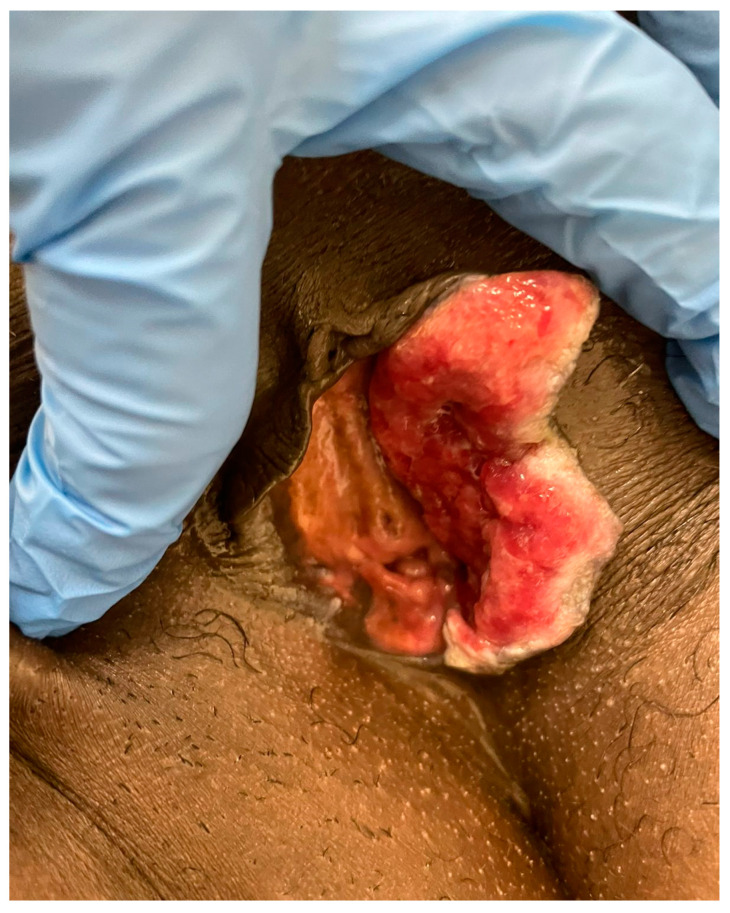
After 1 cycle of carboplatin/paclitaxel.

**Figure 3:**
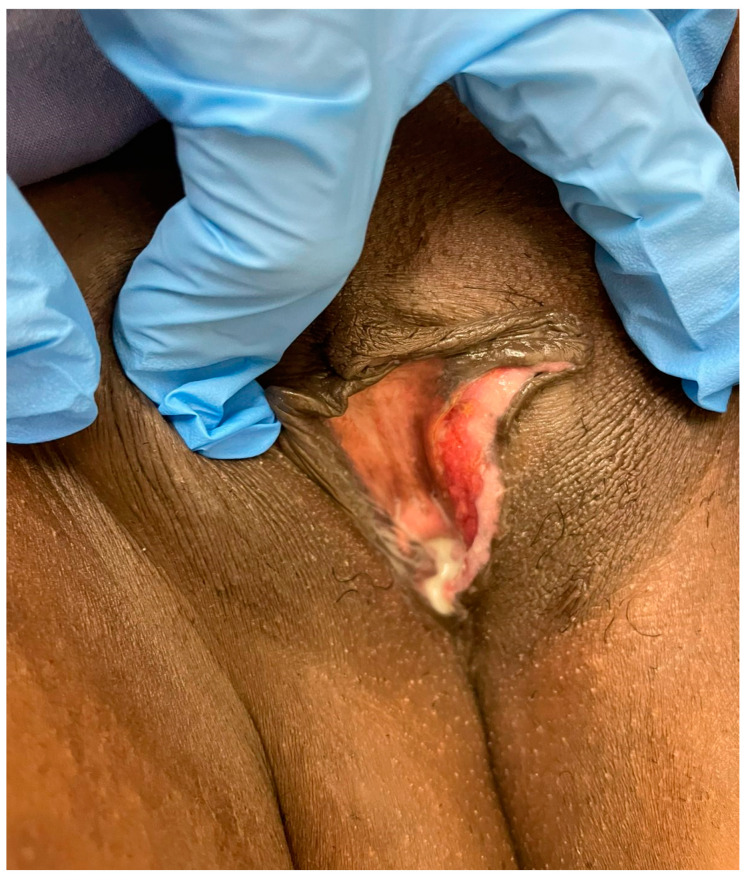
After 3 cycles of carboplatin/paclitaxel.

Ideally, the decision to use NACT should be based on optimising patients for complete surgical excision. An example is shown below (Figure 1, Figure 2 and Figure 3): a young woman with locally advanced vulvar cancer, with close proximity to the anus. She had such a marked response to two cycles of carboplatin and paclitaxel chemotherapy that her tumour was rendered resectable without a posterior exenteration.

The small case series, using various combinations of bleomycin, vincristine, mitomycin C, methotrexate, lomustine, 5-fluorouracil, paclitaxel, carboplatin, and cisplatin, report response rates of 56–67% [8]. 5-FU and cisplatin have been used, with response rates of 20–100% [33,34].

The combination of carboplatin and paclitaxel, as used in other gynaecological malignancies, has been evaluated with or without ifosfamide. Amant et al. demonstrated that patients with locally advanced vulvar cancers treated with 3-weekly carboplatin and paclitaxel, responded sufficiently to be able to undergo surgery [35].

A pooled analysis showed that patients treated with NACT, definitive chemotherapy, or chemoradiation who underwent surgery had a significantly better 5-year overall survival (67% vs. 29%, *p* = 0.001) [36].

### 4.5. Targeted Therapies

It is hoped that the development of targeted therapies will be able to decrease the morbidity associated with radical surgical procedures and radiotherapy and increase the systemic treatment options for women with advanced vulvar cancer. In the current era of advances in molecular biology and individualised approaches to the treatment of cancer, it is recognised that, while all vulvar squamous carcinomas have been treated the same in the past, they do in fact arise from two distinct disease processes, with two distinct causes: one associated with HPV, the other HPV-independent.

Targeted therapies that are under development and investigation, and which may be relevant in the treatment of vulvar SCC, include:

- General treatments that target the “molecular machinery involved in aberrant cell-cycle activity” [37], and
- Other treatments that may be specific to the individual pathogenesis of either HPV-associated or HPV-independent tumours.

(1) Targeted therapies of general relevance to squamous cell carcinomas

The dysregulation of tumour suppressor functions of p53 and Rb, as well as overexpression of Cyclin D1, leads to uncontrolled cell proliferation in both HPV-associated and non-HPV-associated squamous carcinomas of the vulva, and targeting of protein products and downstream pathways may be useful in the treatment of these cancers [37]. Mdm2 is an inhibitor of p53 and has increased expression in many cancers, and Mdm2 antagonists, such as RG7112, are a potential therapy [38]. Ribonucleotide reductase (an enzyme stimulated by aberrant p53 signalling) inhibitors such as Triapine are under investigation [37].

Rapamycin can inhibit pRb phosphorylation and accelerate cyclin D1 and may be used to cause cell-cycle arrest in VSCC, but rapamycin and mammalian target of rapamycin (mTOR) inhibitors have not yet been investigated as treatments for VSCC [37].

Tyrosine kinase proteins are regulators of cellular activity and have been investigated for their role in the aetiology of vulvar cancers, especially C-Kit and epidermal growth factor receptor (EGFR) [37]. There are reports of responses of both locally advanced and metastatic vulvar SCC to Erlotinib (Tarceva), an anti-EGFR tyrosine kinase inhibitor [39,40].

The vascular endothelial growth factor (VEGF) pathway regulates angiogenesis and may be an important factor in the progression of VIN to SCC, as well as a prognostic factor in established SCC [37]. Bevacizumab, a monoclonal antibody directed against VEGF, has been used as an adjunct in the treatment of many gynaecological malignancies and has been used successfully to treat advanced vulvar cancers in combination with carboplatin and paclitaxel chemotherapy [41].

(2a) HPV-Associated Vulvar SCC

HPV is a small double-stranded DNA virus and is composed of more than 100 types of protein, which are sub-divided according to the timing of cell cycle expression into early (E1, E2, E5, E6 and E7) and late (L1 and L2) subgroups [42]. Following infection and viral integration of oncogenic strains of HPV (types 16, 18, 31 and 33), the E6 and E7 oncoproteins inactivate the p53 and pRb tumour suppressors, which then can lead to squamous intra-epithelial lesions (SIL) and, eventually, squamous carcinoma [37].

Vulvar high-grade SIL has a high ki-67 proliferative index and stains diffusely with p16, and the host immune response plays a vital role in both development and progression of HPV-associated vulvar SCC. There is a high prevalence of HPV in pre-cancerous lesions (80%) compared with invasive lesions (40%), and this suggests that there is a better host immune response to vulvar HSIL [43] As cancers that originate in vulvar HSIL have a better prognosis (83% 5-year survival as compared to 48% for HPV-negative tumours) [44], as well as demonstrating an improved response to radiotherapy [45], exploiting the Toll-like receptor (TLR)-mediated host immune response to infection with HPV may be a therapeutic strategy [46,47,48] In preclinical studies, the COX-2 inhibitor celecoxib has been shown to decrease cell growth when used in combination with cisplatin [49], and imiquimod, a topical immune-modulator, is known to be effective in clearing small vHSIL lesions [50,51].

Other immune-based treatments are under investigation in gynaecological malignancies [52]: programmed death 1 (PD-1) is a T-cell coinhibitory receptor that acts as an immune regulator and can contribute to the ability of a tumour to evade immunosurveillance. Pembrolizumab is a monoclonal antibody that prevents interaction between PD-1 and its ligands, PD-L1 and PD-L2. PD-1 has been investigated as a therapeutic target in advanced cervical and vulvar cancer in the phase 1b KEYNOTE-028 trial, and it appeared to be fairly well tolerated but with limited efficacy in women with PD-L1 positive advanced vulvar cancer, with only one woman of 18 achieving a partial response, the rest having stable disease or disease progression [53].

Nivolumab is a PD-1 immune checkpoint inhibitor that has shown limited efficacy in the treatment of a small cohort of women with recurrent/metastatic cervical, vaginal, or vulvar cancers in the CheckMate 358 study [54].

Therapeutic vaccines that target the E6 and E7 oncogenes are also under investigation [37]. Many therapeutic vaccines have been evaluated in clinical trials in HPV-associated cancers, with limited success.

In addition, given the overexpression of p16 in HPV-associated SCC, P16^INK^ inhibitors may be a potential therapy. Targeting of the P16^ink4a^ cellular protein with a P16^ink4a^ vaccine peptide is a therapeutic strategy that aims to inhibit abnormal virus-driven cellular activity [37].

(2b) HPV-independent Vulvar SCC

Up to two-thirds of women with non-HPV related cancers of the vulva have somatic mutations. High mutation rates in PTEN and TP53 are found in differentiated VIN lesions, which are thought to be the immediate predecessor to HPV-independent vulvar SCC. dVIN is thought to be a transient condition, and rapidly progresses to invasive disease [55].

Therapies targeting abnormal P13K/AKT/mTOR signalling activity that results from P1K3CA mutations have shown some encouraging results in pre-clinical studies in vulvar cell lines that have these mutations [56]. MEK inhibitors that target dysregulated RAS oncogenes are also under investigation [37].

Some cyclins and cyclin-dependent kinases (CDK) have been implicated in malignant carcinogenesis in vulvar disease: cyclin D1 overexpression has been reported in HPV-independent vulvar SCC and is associated with depth of invasion and the presence of regional metastases [37].

Next-generation sequencing may identify new genomic targets in HPV-independent vulvar SCC, which is important, as these tumours have a worse prognosis than HPV-associated disease [57].

### 4.6. Recurrent Disease

Management of recurrent vulvar cancer is challenging and should ideally be carried out by a multidisciplinary team. In order to decide on treatment, the sites of recurrence, performance status of the patient, and the previous treatments which have been used should all be taken into account. The patient should have staging investigations repeated in order to determine the precise extent of tumour recurrence. Options for treatment include surgery, radiotherapy, chemotherapy, immunotherapy (if available), and best supportive care.

## 5. Psychosexual Support and Management of Treatment Complications

All patients undergoing treatment for cancer should have access to psychosocial support and counselling from the time of diagnosis and throughout their treatment. This is particularly important for women undergoing treatment for vulvar cancer, where the psychosexual consequences of the disease and its treatment are immense. This is in addition to appropriate and timely information and management of side effects of treatment such as lymphoedema and altered bowel and/or bladder function.

## 6. Areas for Future Research

- A large prospective multicentre study is required to evaluate the outcomes of women treated with neoadjuvant chemotherapy.
- Targeted therapies for HPV-associated and HPV-independent vulvar cancer.
- Therapeutic HPV vaccines.
- Further clinical studies on immune checkpoint inhibitors in women with vulvar SCC.

## 7. Conclusions

Although advanced vulvar cancers account for approximately 30% of presentations of a rare malignancy, their incidence is not negligible and is increasing, especially in young women. While effective treatment modalities, particularly radical surgical excision, are available for early disease, leading to excellent long-term survival rates, patients often suffer functional and psychosexual consequences from their treatment.

The side effects of the ultra-radical surgery and chemoradiation that are required to treat more advanced disease are potentially even greater, and there are many exciting developments in the field of targeted therapies for this condition.

Whatever the management strategy, the underlying principles of management by a multidisciplinary team, individualisation of treatment, and availability of psychosexual support are vital for the holistic care of women with vulvar cancer.

Written consent was obtained from the patient to allow use of her clinical images for this review.

## Figures and Tables

**Table 1:** 2021 FIGO Staging of Vulvar Carcinoma [4].

Stage	Description
I	Tumour Confined to the vulva
IA	Tumour size ≤2 cm and stromal invasion ≤1 mm ^a^
IB	Tumour size >2 cm or stromal invasion >1 mm ^a^
II	Tumour of any size with extension to lower one-third of the urethra, lower one-third of the vagina, lower one-third of the anus with negative nodes
III	Tumour of any size with extension to upper part of adjacent perineal structures, or with any number of nonfixed, nonulcerated lymph nodes
IIIA	Tumour of any size with disease extension to upper two-thirds of the urethra, upper two-thirds of the vagina, bladder mucosa, rectal mucosa, or regional lymph node metastases ≤5 mm
IIIB	Regional ^b^ lymph node metastases >5 mm
IIIC	Regional ^b^ lymph node metastases with extracapsular spread
IV	Tumour of any size fixed to bone, or fixed, ulcerated lymph node metastases, or distant metastases
IVA	Disease fixed to pelvic bone, or fixed or ulcerated regional ^b^ lymph node metastases
IVB	Distant metastases

^a^ Depth of invasion is measured from the basement membrane of the deepest, adjacent, dysplastic, tumour-free rete ridge (or nearest dysplastic rete peg) to the deepest point of invasion. ^b^ Regional refers to inguinal and femoral lymph nodes.

## Abbreviations

CCHR	Concurrent chemoradiation
CI	Confidence interval
C-Kit	Proto-oncogene C-Kit, a tyrosine kinase protein
CT	Computerised tomography
dVIN	Well-differentiated vulvar intraepithelial neoplasia
FIGO	International Federation of Gynaecology and Obstetrics
FNAB	Fine needle aspiration biopsy
Gy	Gray
HPV	Human papillomavirus
HR	Hazard ratio
ICI	Immune checkpoint inhibitors
IMRT	Intensity-modulated radiation therapy
LAVC	Locally advanced vulvar cancer
Mdm2	Murine double minute 2 gene
MRI	Magnetic resonance imaging
mTOR	Mammalian target of rapamycin
NACT	Neoadjuvant chemotherapy
E6/E7	HPV oncogenic proteins E6 and E7
EGFR	Epidermal growth factor receptor
p53	p53 tumour suppressor gene
PET	Positron emission tomography
PD-1	Programmed death-1
PD-L1	Programmed death-ligand 1
PD-L2	Programmed death-ligand 2
pRb	Rb tumour suppressor gene
RCT	Randomised controlled trial
SCC	Squamous cell carcinoma
SIL	Squamous intraepithelial lesion
TLR	Toll-like receptor
VEGF	Vascular endothelial growth factor
VHSIL	Vulvar high-grade squamous intraepithelial lesion
VMAT	Volumetric arc therapy
VSCC	Vulvar squamous cell carcinoma
